# Diet Quality and Its Association with Lifestyle and Dietary Behaviors among Croatian Students during Two COVID-19 Lockdowns

**DOI:** 10.3390/foods12132532

**Published:** 2023-06-29

**Authors:** Danijela Pfeifer, Josip Rešetar, Martin Šteković, Magdalena Czlapka-Matyasik, Donatella Verbanac, Jasenka Gajdoš Kljusurić

**Affiliations:** 1 Faculty of Food Technology and Biotechnology, University of Zagreb, 10000 Zagreb, Croatia; danijela.pfeifer@gmail.com; 2 Faculty of Pharmacy and Biochemistry, University of Zagreb, 10000 Zagreb, Croatia; 3 Department of Human Nutrition and Dietetics, Poznan University of Life Sciences, 60-624 Poznan, Poland; magdalena.matyasik@up.poznan.pl

**Keywords:** COVID-19, lockdown, students, diet quality, dietary behavior, lifestyle behavior, body mass index, nutrition knowledge

## Abstract

The present study aims to assess the diet quality, the relationship between diet quality and lifestyle, and the association of diet quality with body mass index and students’ field of study during COVID-19 lockdown periods (spring and winter) in 2020. Datasets were collected via an anonymous online self-reported questionnaire distributed during two time periods using social media. A total of 1939 Croatian students (82.4% women and 17.6% men) completed the questionnaire. Diet quality was assessed using the pro-healthy diet index (pHDI) and non-healthy diet index (nHDI). An increase in diet quality was noted during both lockdown periods but was lower during the winter lockdown. Cooking for oneself was associated with a high level of pHDI, while ordering or buying ready-to-eat food was linked to a low level of pHDI. Additionally, a decrease in screen time and increased physical activity was associated with high pHDI values. Furthermore, during both lockdown periods, students with a BMI above 30 kg/m^2^ had the highest nHDI values compared to other students. Although positive changes were found during both lockdown periods, they were less pronounced throughout the winter lockdown. Further studies are needed to elucidate the real impact of these changes in the post-COVID period.

## 1. Introduction

In response to the outbreak and rapid spread of COVID-19 (coronavirus disease 2019), including the increasing death rate, the World Health Organization declared the COVID-19 pandemic on 12 March 2020 [1]. To date, more than 760 million people have been infected, and over 6.9 million have died, globally due to the COVID-19 disease. In the Republic of Croatia, infection has been confirmed in more than 1.3 million cases, while almost 18 thousand have died due to complications related to COVID-19 [2].

The relaxation of the global lockdown, introduced on 19 March 2020 [3], began in late April of the same year. Despite the improvement of the epidemiological situation during the summer of 2020, the spread of the SARS-CoV-2 virus (Severe Acute Respiratory Syndrome Coronavirus 2) started again in autumn, which led to the introduction of new measures on 28 November 2020. Although complete lockdown has not been introduced, restaurants, cafes, and gyms have again been closed, and gatherings have been restricted, while universities reintroduced online lectures. The abolition of contact lectures, more time spent indoors, the lack of social interaction, and the forced customization to an entirely new way of fulfilling student obligations have been reflected in the average student’s numerous habits and behavior patterns.

The COVID-19 pandemic brings upon a potential deterioration of the global obesity pandemic we have been battling for decades [4]. An increase in sedentary lifestyle due to lockdown, reported in previous research [5,6], can lead to an imbalance in energy expenditure and, consequently, an increase in body weight. Additionally, it has been shown that there is a significant difference in the severity of symptoms of COVID-19 disease depending on body mass index [7]. Furthermore, in terms of cardiometabolic health during the COVID-19 lockdown, besides body weight, the connection between diet and the immune system was also emphasized, as well as regular physical activity and adequate quality and quantity of sleep [8].

With universities’ closure, the entire teaching program was moved online. In order to fulfill their academic obligations, students were forced to spend more time in front of the screen, which was also noted in the population of adolescents in Croatia [6]. Spending six to eight hours a day in front of a screen is considered an independent factor in the development of obesity [9,10]. Additionally, it was reported that during the COVID-19 pandemic, poorer diet quality was observed in individuals who had significantly increased their screen time [11].

On the other side, the COVID-19 lockdown was also associated with some beneficial changes. An increase in cooking frequency and the time and effort spent on food preparation were recorded and positively associated with higher diet quality during COVID-19 [12,13,14]. Additionally, decreases in alcohol [15] and fast-food intake [13,16] have been reported.

In this study, three parameters were accurately investigated: (i) diet quality, (ii) the relationship between diet quality and lifestyle, and (iii) the association of diet quality with body mass index and students’ field of study. We assessed these before the lockdown, and during the spring and winter of 2020—lockdowns in the Republic of Croatia.

## 2. Materials and Methods

### 2.1. Study Design and Participants

This research was conducted as a part of the UNI-COVID project, which included Polish, Spanish, Portuguese, Ukrainian, Turkish, and Croatian students with no exclusion criteria. This project is an observational cross-sectional study that used a structured questionnaire developed using the Google Forms tool (Supplementary Materials). Students attending all Croatian universities (undergraduate, graduate, and postgraduate level of study) were invited to participate in this study and fill out the questionnaire via the official e-learning platform of the University Computing Centre—Merlin. The questionnaire contained questions about students’ sociodemographic characteristics (gender, age, marital status, place of residence, level of education, and household’s overall situation), study field, eating habits, lifestyle, and mental state both before and during the spring and winter lockdowns, and anthropometric characteristics (body weight and body height). The questionnaire provided respondents with complete anonymity.

This research is in line with the ethical standards of the institutional and national committee and the Helsinki Declaration. Students agreed to participate in the research via the digital form for informed consent. Since this study does not belong to a medical experiment, it was exempt from ethical approval from the Poznan University of Medical Sciences Bioethics Committee according to Polish laws and Good Clinical Practice (GCP) regulations (decision number: 527/20). The questionnaire consisted of questions taken from a KomPAN questionnaire [17].

The questionnaire was taken on two occasions. It was first implemented during the spring lockdown 2020—it was available for fulfillment from 18 May to 7 June 2020. In that period, responses were collected from 751 students (156,325—number of all Croatian students; 0.5% response rate). It was conducted for the second time during the winter lockdown in 2020, and 1188 students (155,627—number of all Croatian students; 0.8% response rate) completed the questionnaire from 14 to 22 December 2020. Sociodemographic characteristics of students included age, gender, marital status, residence, level of education, and the general situation in the household.

### 2.2. Dietary and Lifestyle Practices Evaluation

Students answered questions about dietary and lifestyle behaviors before introducing lockdowns and whether those behaviors changed during the lockdowns. Lifestyle behaviors included screen time, sleeping time, cooking habits, food ordering frequency, the daily number of meals, their time of consumption, and the level of physical activity.

Questionnaire part B, on the frequency of consumption of certain foods, was used to assess the quality of nutrition before the introduction of lockdowns. The structure of questionnaire part B is briefly described by Pfeifer et al. [18].

Students marked the frequency of consumption of certain foods by choosing one of six frequency categories—from never to several times a day. They also assessed the change in the frequency of consumption for 22 food groups by choosing for each group: “more”, “less”, or “equal”, in relation to consumption before the introduction of lockdowns.

### 2.3. Diet Quality Evaluation

Each of the six categories of food consumption frequencies was recalculated to the daily frequency as follows: (1) never → 0 times a day, (2) 1–3 times a month → 0.06 times a day, (3) once a week → 0.14 times a day, (4) several times a week → 0.5 times a day, (5) once a day → 1 time a day and (6) several times a day → 2 times a day.

Two indices were used to assess the diet quality—pHDI (pro-Healthy Diet Index) and nHDI (non-Healthy Diet Index). For the period before the lockdowns, indices were calculated as a sum of recalculated daily frequencies of respectable corresponding food groups as described in the previous papers [17,18].

The pHDI and nHDI were expressed as the scored and total possible points ratio. The indices were categorized using an a posteriori approach. Three levels were defined according to the tertiary distribution:

(a) pHDI: lower tertile (<23.9%), middle tertile (23.9–38.3%), and upper tertile (≥38.3%).
(b) nHDI: lower tertile (<8.2%), middle tertile (8.2–14.4%), and upper tertile (≥14.4%).

During the lockdown period, students assessed the change in their consumption frequency for each food group. For a student who increased their consumption of a certain food group, the number of points rose to the points for the corresponding frequency category above the one marked for the period before the lockdowns. Respectively, for a student who decreased their intake, a corresponding number of points was awarded for the frequency category below the one indicated for the period before the lockdowns were introduced. The sum for both indices was calculated and categorized the same way as before the measures. For each index, three levels were defined according to the tertiary distribution:

(a) pHDI: lower tertile (<27.6%), middle tertile (27.6–42.1%), and upper tertile (≥43.1%).
(b) nHDI: lower tertile (<8.9%), middle tertile (8.9–16.8%), and upper tertile (≥16.8%) [18].

### 2.4. Statistical Analysis

All of the data obtained by the questionnaire were qualitative, except for students’ body height, body weight, and age. Qualitative data for the frequency of certain food groups’ consumption were converted and coded into quantitative data (pHDI and nHDI). The F-test was used to check the equivalence of the variances between the two datasets, while the statistical significance of the difference was tested by the Student’s *t*-test and χ^2^-test for categorical variables. A box plot diagram was used to show the correlation between pHDI, nHDI values, and body mass index groups (<18.5; 18.5–24.9; 25–29.9; and ≥30 kg/m^2^), and to show the pHDI and nHDI of individual scientific fields of study. The sample size was calculated as described by Pfeifer et al. [18].

Furthermore, descriptive statistics were used to show the general characteristics of students depending on the levels of pHDI during the lockdowns and the changes in dietary and lifestyle behaviors during the lockdowns. For the data relating to the time before the lockdowns, descriptive statistics were used depending on the levels of pHDI before the introduction of the lockdowns. MS Office Excel 2016 and SPSS v. 17 (SPSS Inc., Chicago, IL, USA) were used for all of the above data processing.

## 3. Results

The distribution by age, gender, and body mass index (BMI) of students during the spring (response rate 0.5%) and winter lockdowns (response rate 0.8%) is shown in Table 1 [18]. Students also provided information on change of residence during the lockdowns and reported potential employment status changes. Using the self-estimated body weight and body height data, the BMI was calculated as the ratio of body weight expressed in kilograms and the square of body height in meters.

Table 2 shows pHDI and nHDI values before and during lockdowns for spring and winter 2020. During the spring lockdown, pHDI values significantly increased (*p* < 0.001) compared to pHDI values before the lockdown. A similar change was observed by repeating the questionnaire in winter 2020—a statistically significant increase (*p* < 0.001) in pHDI values during the lockdown. Additionally, a statistically significant difference was noted between pHDI values during the spring and winter lockdowns—pHDI values were higher during the spring lockdown.

Regarding the nHDI values, statistically significant increases (*p* < 0.001) compared to the periods before the lockdowns were observed during both lockdowns (Table 2). The nHDI values before the spring lockdown were significantly lower than before the winter lockdown. The same trend was observed during the lockdowns, and the nHDI values during the winter lockdown were significantly higher (Table 2).

Table 3 confirms the previously observed difference between pHDI values during the spring and winter lockdowns. The distribution of students according to levels of pHDI shows that during the spring lockdown, the high level was the most common (37.6%) compared to the low and medium levels. During the winter lockdown, a different trend was noted—the share of the total number of students decreased with increasing pHDI.

The χ^2^-test showed that students who lived alone or with a partner most often belonged to the low level of pHDI. However, those who lived with a partner were more likely to belong to the high level than students who lived alone (31.9 vs. 26.2%) (*p* < 0.05).

Although not statistically significant, women were more likely than men to have a high level of pHDI—38.6 vs. 29.1% during the spring and 28.7 vs. 21.5% during the winter lockdown. Furthermore, during spring and winter lockdowns, students in cities with more than 100,000 inhabitants, compared to other residences, most often had a medium level of pHDI, while students residing in villages or cities with less than 20,000 inhabitants most often had a high level of pHDI. During both lockdowns, students under the age of 20 most often had a low level, those aged 25–30 years had a medium level, and students with a master’s degree had a high level of pHDI (Table 3).

**Figure 1:**
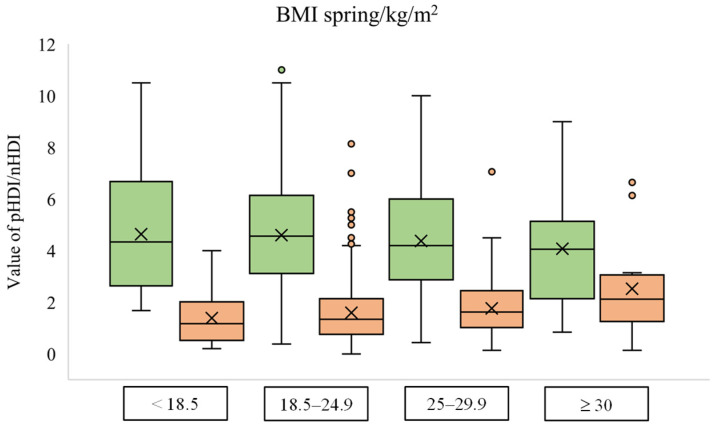
Box plot diagram of pHDI (green) and nHDI (orange) values according to the body mass index group during the spring lockdown.

During the spring lockdown, a declining trend in diet quality with an increase in body mass index was observed (Figure 1). The group with the lowest BMI has the highest mean pHDI (4.64 ± 2.26). As the BMI increased, the mean pHDI decreased, and the lowest mean pHDI was noted for students with a BMI above 30 kg/m^2^ (4.07 ± 2.18). Although the mean pHDI for the group with BMI values from 18.5 to 24.9 kg/m^2^ was lower than for the group with lower BMI, the median pHDI for the normal weight group was 4.56, while the mean pHDI for malnourished students was 4.34. On the other hand, both mean values and medians of nHDI increased with increasing BMI—the lowest nHDI was noted for students with a BMI of less than 18.5 kg/m^2^ (1.39 ± 1.03), while the highest nHDI was detected in obese students (2.52 ± 1.81).

**Figure 2:**
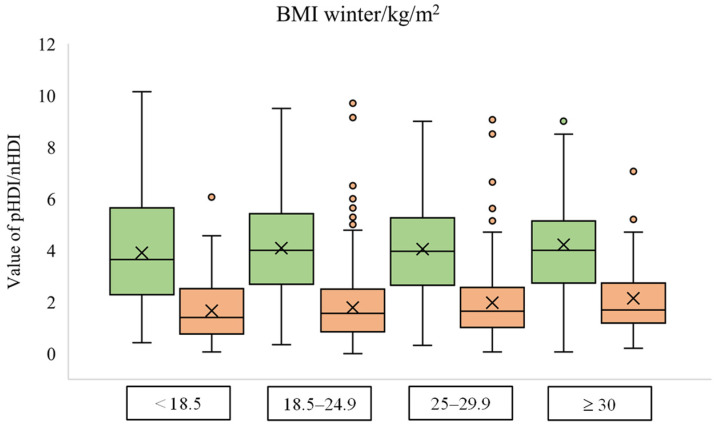
Box plot diagram of pHDI (green) and nHDI (orange) values according to the body mass index group during the winter lockdown.

The dependency between diet quality and body mass index during the winter lockdown shows equal relation for nHDI but not for pHDI. Students with the lowest BMI have the lowest nHDI (1.66 ± 1.19), while students with the highest BMI have the highest nHDI (2.14 ± 1.47). On the other hand, pHDI values were similar in all BMI groups, with the result approximately 4 (Figure 2).

Students’ characteristics (age, BMI, and gender) according to their fields of study are presented in Table S2.

**Figure 3:**
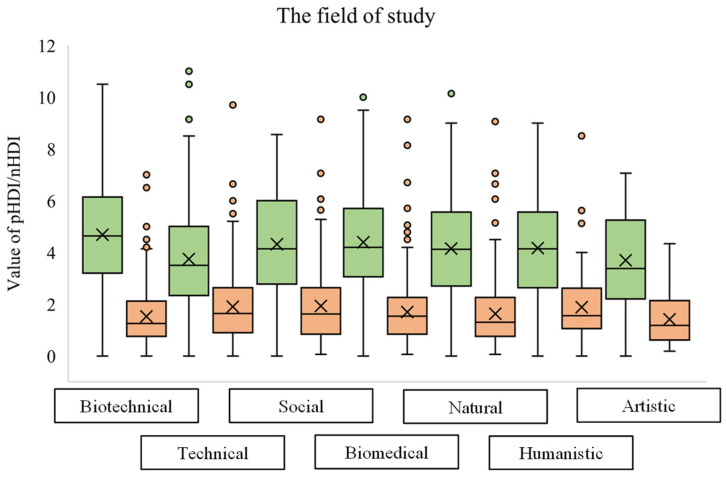
Box plot diagram of pHDI (green) and nHDI (orange) values during the spring and winter lockdowns according to the field of study. (Students who did not define their field of study (5 students) are not shown in the diagram).

Depending on the field of study, the mean values of pHDI and nHDI differ (Figure 3). The mean pHDI values ranging from the highest to the lowest were biotechnical sciences > biomedicine and health > social sciences > humanistic sciences > natural sciences > artistic studies > technical sciences. On the other hand, nHDI values, from the lowest to the highest mean value, ranged according to the following sequence: artistic studies < biotechnical sciences < natural sciences < biomedicine and health < humanistic sciences < technical sciences < social sciences. Although they had one of the highest mean values of pHDI, students of the social sciences also had the highest mean value of nHDI. Students of the technical sciences had a similar mean value of nHDI, but they also had the lowest mean value of pHDI. On the other hand, in addition to their relatively low mean value of pHDI, students of artistic studies also had the lowest mean value of nHDI compared to the other fields of study. Students from biotechnical sciences had the highest cumulative diet quality—the largest difference between the mean value of pHDI and nHDI. In second place were students whose studies belong to the field of biomedicine and health.

During the two round of lockdowns, significant differences (*p* < 0.05) were confirmed in the values of pHDI [18]; therefore, students’ lifestyles and changes distributed are presented only according to levels of pHDI (Table 4 and Table 5).

The distribution into three a posteriori levels of pHDI was more even before the lockdowns compared to the period during the lockdowns. Before and during the spring lockdown, the group of high pHDI is the most common. On the other hand, before the winter lockdown, the medium level is the most common, in contrast to the distribution during the winter lockdown, where the low level of pHDI dominated (Table 4 and Table 5).

There was a statistically significant (*p* < 0.05) difference between the groups of certain lifestyle characteristics, both before the spring and winter lockdowns. Thus, 42.4 and 37.8% of students who cooked for themselves before the spring and winter lockdowns, respectively, had a high level of pHDI, while 41.8 and 41.0% of students who mainly ordered or bought ready-to-eat food before the spring and winter lockdowns, respectively, had a low pHDI level. Students who predominantly ordered food had the lowest propensity for high pHDI. Furthermore, prior to the introduction of lockdowns, students who had high physical activity at work and in their free time predominantly had a high level of pHDI. Of the total number of highly active students, 53.1 and 53.6% had the high pHDI before the spring lockdown, while the analogous percentages before the winter lockdown were 43.9 and 42.8%. On the other hand, students with low physical activity most often had a low level of pHDI (Table 4).

There was a significant difference (*p* < 0.05) in pHDI level between students with different places of residence and working status before the introduction of winter but not before spring lockdown (Table 4). Thus, roommates in an apartment before the winter lockdown had predominantly low, while those living in the family home or alone had a medium level of pHDI. Furthermore, students who worked part-time before the winter lockdown mostly had high pHDI (41.5%).

Although there was no statistically significant difference, students with less time spent in front of the screen before introducing a lockdown tended to have a higher level of pHDI. Additionally, students who ordered food once a week or more tended to have a lower level of pHDI, as did those who did not consume a single meal at a usual time before the lockdowns (Table 4).

Consistent with the results from before the spring and winter, during the lockdowns, students who increased their physical activity, either at work or in their spare time, predominantly had a high level of pHDI. Students in the high pHDI group increased physical activity at work and in leisure time more significantly during the spring lockdown than during the winter lockdown (45.4 and 44.33% vs. 35.2 and 35.7%, respectively). Additionally, students who did not have a habit of consuming meals at regular times during the spring and winter lockdowns predominantly had a low pHDI level, while those who consumed all meals at normal times were more likely to have a high level of pHDI during the lockdowns (Table 4 and Table 5).

In contrast to the period during the winter lockdown, students who increased the frequency of ordering food had a predominantly low pHDI, while students who reduced or maintained the same frequency during the spring lockdown tended to have a high level of pHDI. The same trend during the spring lockdown was also observed for body weight change—students who increased their body weight were more likely to have low pHDI. Regarding the change in cooking habits, students who started cooking for themselves during the spring lockdown had high pHDI (Table 5).

Furthermore, students who lost their jobs during the lockdowns showed a predominantly high level of pHDI during both winter and spring lockdowns. An increase in screen time showed an association with a low level of pHDI during the winter lockdown, while students who reduced screen time tended to have higher pHDI values—results during the spring lockdown showed the opposite effect (*p* > 0.05). During the winter lockdown, both students who increased and those who decreased the number of meals consumed were more likely to have low pHDI, while during the spring lockdown, those who reduced the number of meals predominantly had a high level of pHDI.

## 4. Discussion

An increase in the diet quality during the spring lockdown, compared to the diet quality before, was recorded in 16 European countries, including Croatia [19,20]. The same result was found in the adult population in Canada [21]. To investigate if the same trend reflected in the quality of students’ diet, the students’ diet quality was assessed through two indices—pHDI (pro-Healthy Diet Index) and nHDI (non-Healthy Diet Index) [22]. The pHDI was calculated using white meat, fish and seafood, vegetables, fruits, legumes, and dairy products as the declared intake. On the other hand, the nHDI included the intake of red meat, processed meat products, sweets, and sweetened, carbonated, energy, and alcoholic beverages [18]. Students in Croatia follow the global trend, and during the spring lockdown, an increase of 17.0% was noted for pHDI. During the winter lockdown, students also increased their pHDI, but the increase was lower—7.3%. Although there was an increase in the positive, the negative aspect of the diet also increased during the spring and winter lockdowns (by 14.7% and 13.8%, respectively).

Although no statistically significant effect of gender on pHDI was found, it was observed that female students were more likely to have higher pHDI than male students (Table 3). Grzymisławska et al. [23] proposed a link between gender and diet quality, where women are more engaged in controlling their body weight and choosing healthier foods. At the same time, men prefer fatter meals with more robust flavors. During the spring lockdown, COVIDiet study in Croatia indicated a higher quality of women’s diets than men [13]. Both COVIDiet study and Karam et al. [24] suggest an association between greater compliance with the Mediterranean diet and a higher level of education, which was also found in the student population.

During the winter lockdown, students living with a partner were more likely to have higher pHDI values than students living alone. Gustat et al. [25] showed that living in a marriage or with a partner is associated with lower consumption of chips, sweets, and cakes. Additionally, people living in a marriage have a higher frequency of cooking, which is associated with higher diet quality [26], lower daily energy intake, and lower fat and sugar consumption. In contrast, divorce and independent living are associated with a low frequency of cooking [27].

The increased cooking frequency was highlighted as a positive change [28] and studies reviewed by Mignogna et al. [29] were concordant in highlighting an increased preparation of homemade food during the spring lockdown. The results of our study show that students who cooked for themselves before the introduction of lockdowns had a high level of pHDI, which confirms the aforementioned correlation between cooking frequency and higher diet quality. Moreover, the change in cooking habits had a statistically significant effect (*p* < 0.05) on the affiliation to a certain level of pHDI during the spring lockdown. The trend was similar—students who started cooking for themselves during the measures were more likely to have high pHDI values. On the other hand, students who mostly ordered or bought ready-made food had a low level of pHDI, 41.8 and 41.0%, before the spring and winter lockdown, respectively. In contrast to the positive effect of cooking, ordering or buying ready-made food negatively affects diet quality. Moreover, it is associated with the consumption of low nutrient-density foods and a higher intake of saturated fats and sweets [30,31]. Accordingly, students who ordered food once a week or more were more likely to have low pHDI values.

Individuals with excessive body weight and obesity are at greater risk of developing more severe clinical outcomes of COVID-19 disease [7]. People with obesity also have a lower diet quality than those with adequate body weight [32]. In our study, students with a BMI higher than 30 kg/m^2^ had the lowest pHDI and the highest nHDI compared to other students during the spring lockdown. However, it should be borne in mind that these observations could happen only during lockdowns and might not be permanent. Higher BMI values are associated with lower diet quality, lower levels of physical activity, and a higher frequency of overeating during the spring lockdown [33]. Additionally, students who observed weight gain during the spring lockdown were more likely to have a lower pHDI than students who reduced or maintained the same body weight. During the winter lockdown, students with obesity continued to have the highest nHDI, but there was a change in the pHDI scores, where the mean value was similar for all BMI groups. Compared to the results during the spring lockdown, pHDI values decreased for all BMI groups except for the group ≥ 30 kg/m^2^, where the mean value increased. An increase in nHDI values was also noted, again except for the group of students with obesity where, compared to the spring, the mean value decreased during the winter lockdown. These results can be explained by long-term stress and anxiety, which are associated with higher consumption of energy-rich and nutritionally poor foods [34]. For example, 51% of Italian young adults reported a higher consumption of sweets, cakes, and pastry products during the spring lockdown [5]. On the other hand, the communication of the impact of increasingly severe disease symptoms and increased mortality rates by the media may have impacted the diet of students with obesity by highlighting the association of obesity with more severe outcomes of COVID-19 disease.

The pHDI and nHDI values varied according to the students’ field of study, so students from biotechnical sciences and biomedicine and health had the highest pHDI values. In contrast, those from technical sciences had the lowest. Students from biotechnical sciences also had one of the lowest nHDI values, while technical and social sciences students had the highest nHDI values. In a paper by Muñoz-Rodríguez et al., students from the field of biomedicine had a better diet quality in contrast to non-biomedical students [35]. Similar results to ours were found in Polish and Saudi Arabian studies [36,37], where students of nutrition and the health field of study had higher dietary quality (DQI) compared to students of social and humanistic sciences and other faculties. Furthermore, health-literate students having relevant information on proper nutrition were associated with healthier eating behaviors during the spring lockdown [38]. This explains the greater diet quality for biotechnical science students and students from the field of biomedicine and health, and poorer diet quality for technical science students.

Although research shows an inconsistent association between mealtimes and diet quality [39], meal consumption at unusual times of the day was associated with lower diet quality—primarily higher saturated fat intake and lower energy intake from cereals. Additionally, it has been found that people who consume meals at normal times have the highest diet quality [40,41]. Thus, in the student population of Croatia, during the spring and winter lockdowns, meal consumption at a certain time had a statistically significant effect on the level of pHDI (*p* < 0.05). Students who consumed all meals at usual times tended to have a high level of pHDI. At the same time, irregular consumption was associated with a low level of pHDI.

The daily rhythm and adequate amount of sleep appear to be related to the functioning of the immune system at an optimal level [42]. Before introducing the lockdowns, approximately 70% of students in Croatia slept 7 or 8 h. During the spring lockdown, significantly more students increased their sleep time than during the winter lockdown (57.1 vs. 37.8%). No connection between sleep duration and diet quality was noted, but the questionnaire did not cover the quality of students’ sleep. At the time of global lockdown, numerous sleep problems were found [43]; a large number of respondents from northern Italy (43%) had symptoms of insomnia [44], and 37.3% of young adults in Italy reported sleeping worse during the spring lockdown [5]. Moreover, students who slept nearly 8 h a day had better sleep quality. Although 44.7% of students slept more during the spring lockdown, only 15.5% of the total number of students in this study slept better, and 32.1% rated their sleep quality worse than before the lockdown [45].

Spending more time in front of the screen is associated with poorer diet quality and reduced physical activity [10,11,46]. Compliance is also visible in the student population in Croatia, where those with less screen time tended to have a high level of pHDI before the lockdowns. Moreover, during the winter lockdown, the increase in screen time was associated with a low level of pHDI, while students who reduced their screen time were more likely to have higher pHDI values.

On the other hand, increasing physical activity levels could likely reduce the severity of COVID-19 disease symptoms [47]. Before the introduction of lockdowns, slightly more than 50% of students had moderate physical activity, i.e., 50% of time spent actively at work, while in their free time they walked, cycled, or exercised 2–3 h a week, which is in line with the results of Cancello et al. [44]. More than 40% of students increased their physical activity during the spring lockdown, while approximately 30% decreased it. The decrease in physical activity was slighter than the decrease noted by Brancaccio et al. [48], where more than 40% of participants decreased their physical activity. However, the introduction of the winter lockdown seems to have had a more significant effect on reducing students’ physical activity. During the winter lockdown, a decrease was observed in more than 50% of students, while approximately 20% increased their physical activity. This result is probably due to the inability to go to gyms and hold group workouts combined with colder outdoor weather conditions. Although there were differences in the change in physical activity during the spring and winter lockdowns, in both cases, the level of physical activity was associated with high pHDI values. Students with high physical activity predominantly had high pHDI before introducing the lockdowns, while those with low physical activity also had low pHDI values. A similar trend was observed for changes in physical activity during the lockdowns, where students who increased their physical activity had high levels, while a decrease in physical activity was associated with low pHDI. These results are in accordance with those found in the population of young adults in Italy during the spring lockdown. Respondents with low adherence to the Mediterranean diet had a three times higher risk of physical inactivity than respondents with high adherence [5].

Pérez-Rodrigo et al. [49] observed that individuals aged 18–34 years were more eager to increase their physical activity during the spring lockdown, which could explain the dominant increase in physical activity observed in students in Croatia at that time.

Since the students’ diet quality increased during both lockdowns, it is essential for future studies to identify all of the contributors to this phenomenon and find a way to maintain them in the post-COVID period. Additionally, it was recorded that higher diet quality was associated with higher cooking frequency and lower food ordering practice—therefore, it should consider the implementation of students’ cooking workshops as a potent tool to increase diet quality among the student population. In addition, students from biotechnical science and the field of biomedicine and health recorded the highest diet quality—since these subpopulations of students have the greatest knowledge about nutrition, the introduction of basic education regarding nutrition in syllabi of other study fields may be a way to go for providing better student nutrition.

## 5. Limitations

Using an online questionnaire as a tool, in a short time we collected the information of many students without breaking the regulations and recommendations of social distancing. However, some limitations should be acknowledged. A KomPAN questionnaire, from which most questions were taken to create an online questionnaire used in this study, has not been validated in Croatia—but it was used in the study since it best fits the target population, and it allowed the comparison with the results of other countries included in the UNI-COVID project. During spring and winter lockdowns, students who decided to take part in the study were predominantly females (85.6% and 80.4%, respectively), untrained, and could not ask for explanations if they had any doubts. This might have resulted in under/overestimation of actual food proportion intakes. The students answered questions retrospectively about their diet and lifestyle before the lockdown, which is also a limitation due to memory recollection. Additionally, the self-reported weight and height could have led to bias when BMI was calculated. Transferring qualitative data to quantitative for the assessment of diet quality during lockdowns and restricting the variation in the HDI options could also lead to a potential calculation error. However, it is possible to express nHDI and pHDI values in a scale ranging from 0 to 100 [22,50], instead of tertiles [51], and to determine adherence to a healthy/unhealthy diet (low: <33; medium: 34–67; and high for sum > 67) according to Jezewska-Zychowicz et al. [50]. Therefore, we suggest that before recalculating the indexes (pro-healthy diet index and non-healthy diet index), the authors adapt to one of the two methods listed. It also needs to be pointed out that pHDI and nHDI during the spring and winter lockdown were assessed with the same approach, although the consumption “before” the lockdown is the winter period (before the first round of data collection); furthermore, the autumn period preceded the second round of data collection and it is possible that even in the times preceding the lockdown, there was different consumption from different food groups. However, given the availability in the supply chain, which was very limited, we assume that there were no significant differences, but we can neither confirm nor decline it. Furthermore, the online questionnaire could miss some students who have limited access to digital technologies or who do not use digital technologies frequently. Finally, the online questionnaire may have led to a bias relating to the participation of people who were more interested in or motivated by the study subject.

## 6. Conclusions

The diet quality of students in Croatia generally increased during the spring and winter lockdown. Higher diet quality, during both winter and spring lockdowns, was associated with a decrease in food ordering frequency, a lesser increase in body weight, and an increase in physical activity either at work or in leisure time. As the student’s BMI increased, the diet quality, shown as pHDI, decreased, and the lowest mean pHDI was noted for students with a BMI above 30 kg/m^2^. Regarding the association of diet quality and field of study, students from biotechnical sciences had the highest cumulative diet quality—the largest difference between the mean value of pHDI and nHDI. In second place were students whose studies belong to the field of biomedicine and health.

## Figures and Tables

**Table 1:** Distribution of students during spring and winter lockdowns according to gender, age, and BMI [18].

Lockdown Collection	Students’ Characteristics										
	Gender *			Age (Years)				Body Mass Index (kg/m^2^) *			
	Female	Male	Other	<20	20–24	25–30	<30	<18.5	18.5–24.9	25–29.9	>30
Spring Lockdown											
N = 751	643	103	5	98	542	101	10	42	583	103	23
%	85.6	13.7	0.7	13.0	72.2	13.4	1.3	5.6	77.6	13.7	3.1
Winter Lockdown											
N = 1188	955	219	14	235	855	90	8	86	924	140	38
%	80.4	18.4	1.2	19.8	72.0	7.6	0.7	7.2	77.8	11.8	3.2

* Statistically significant differences (*p* < 0.05) between BMI evaluated by the Student’s *t*-test and between gender evaluated by the χ^2^-test.

**Table 2:** Values of diet quality indices (pHDI and nHDI) before and during spring and winter lockdowns are shown as mean ± standard deviation.

Diet Quality	Spring Lockdown		Winter Lockdown	
	Before	During	Before	During
pHDI ^1^	3.89 ± 1.80	4.55 ± 1.98	3.79 ± 1.74	4.07 ± 1.86 *
nHDI ^2^	1.43 ± 0.92	1.64 ± 1.14	1.59 ± 1.10 *	1.81 ± 1.29 *

^1^ Pro-Healthy Diet Index. ^2^ Non-Healthy Diet Index. * Statistically significant differences (*p* < 0.05) between spring and winter lockdowns’ diet quality indices evaluated by the Student’s *t*-test.

**Table 3:** Students’ general characteristics distributed according to levels of pHDI during the spring and winter lockdowns.

**Students’ Characteristics**	**pHDI**									
	**Spring Lockdown**					**Winter Lockdown**				
	All	Low	Medium	High	*p* *	All	Low	Medium	High	*p* *
	N	%	%	%		N	%	%	%	
	751	31.4	31.0	37.6		1188	39.9	32.7	27.4	
Gender **					0.183					0.052
Female (%)	643	30.8	30.6	38.6		955	38.6	32.7	28.7	
Male (%)	103	35.9	35.0	29.1		219	46.1	32.4	21.5	
Marital status **					0.892					**0.014**
Single (%)	580	31.0	32.1	36.9		779	39.0	34.8	26.2	
With a partner (%)	78	35.9	28.2	35.9		367	42.2	25.9	31.9	
Married (%)	4	25.0	25.0	50.0		11	45.5	54.5	0.0	
Prefer not to say (%)	87	29.9	27.6	42.5		30	33.3	50.0	16.7	
**Place of residence** (number of citizens)					0.276					0.376
City > 100,000 (%)	288	31.3	35.4	33.3		500	38.4	34.8	26.8	
City 20,000–100,000 (%)	148	32.4	29.7	37.8		202	45.5	31.7	22.8	
City < 20,000 (%)	147	34.7	27.2	38.1		212	40.6	30.2	29.2	
Village (%)	168	28.0	28.0	44.0		274	38.0	31.4	30.7	
**Age** (years)					0.880					0.314
<20 (%)	98	34.7	31.6	33.7		235	42.1	32.8	25.1	
20–24 (%)	542	30.6	30.4	38.9		855	39.8	31.8	28.4	
25–30 (%)	102	32.4	34.3	33.3		90	33.3	42.2	24.4	
>30 (%)	9	33.3	22.2	44.4		8	62.5	12.5	25.0	
**Level of education**					0.909					0.205
Upper secondary (%)	444	31.1	31.8	37.2		772	41.6	32.8	25.6	
Bachelor’s degree (%)	263	31.2	30.8	38.0		364	36.5	33.5	29.9	
Master’s degree (%)	44	36.4	25.0	38.6		52	38.5	25.0	36.5	
**Household’s overall situation**					0.139					0.296
Very modestly (%)	8	50.0	25.0	25.0		14	50.0	21.4	28.6	
Modestly (%)	43	34.9	30.2	34.9		93	41.9	34.4	23.7	
Normally (%)	545	29.5	33.4	37.1		833	40.3	34.0	25.7	
Relatively wealthy (%)	140	38.6	22.9	38.6		212	36.3	27.4	36.3	
Very wealthy (%)	5	20.0	0.0	80.0		3	33.3	33.3	33.3	
Prefer not to say (%)	10	10.0	40.0	50.0		33	42.4	33.3	24.2	

* *p* represents *p*-value between the groups evaluated by the χ^2^-test. *p*-values < 0.05 are highlighted in bold. ** There were 5 students during spring lockdown and 14 students during winter lockdown who declared their gender as other, while 1 widowed and 1 divorced during spring lockdown and 1 divorced during winter lockdown were excluded in order not to weaken the interpretability of the χ^2^-test.

**Table 4:** Students’ lifestyles distributed according to levels of pHDI before the spring and winter lockdowns.

**Students’ Lifestyle**	**pHDI**									
	**Spring Lockdown**					**Winter Lockdown**				
	All	Low	Medium	High	*p* *	All	Low	Medium	High	*p* *
	N	%	%	%		N	%	%	%	
	751	33.3	31.0	35.7		1188	33.4	35.9	30.6	
**Place of residence before lockdown**					0.749					**0.048**
Family home (%)	293	34.5	29.7	35.8		615	31.1	38.5	30.4	
Alone (%)	105	28.6	36.2	35.2		130	33.1	38.5	28.5	
Students’ residence (%)	169	36.1	31.4	32.5		199	30.7	34.7	34.7	
Shared flat (%)	184	31.5	29.9	38.6		244	41.8	29.1	29.1	
**Working status before lockdown**					0.862					**0.018**
Half-time (%)	142	31.7	34.5	33.8		176	29.0	29.5	41.5	
Full-time (%)	62	33.9	27.4	38.7		76	31.6	36.8	31.6	
I do not work (%)	547	33.6	30.5	35.8		936	34.4	37.1	28.5	
**Screen time before lockdown** (hours)					0.529					0.517
<2 (%)	84	32.1	28.6	39.3		132	28.8	35.6	35.6	
2–4 (%)	304	32.9	28.9	38.2		434	32.5	35.9	31.6	
4–6 (%)	222	34.2	31.1	34.7		325	33.5	36.6	29.8	
6–8 (%)	84	38.1	35.7	26.2		187	38.5	36.4	25.1	
8–10 (%)	39	20.5	43.6	35.9		72	27.8	37.5	34.7	
>10 (%)	18	38.9	27.8	33.3		38	44.7	26.3	28.9	
**Sleeping time before lockdown** (hours)					0.373					0.465
<4 (%)	2	0.0	50.0	50.0		5	80.0	0.0	20.0	
4–5 (%)	15	40.0	13.3	46.7		19	31.6	47.4	21.1	
5 (%)	17	29.4	35.3	35.3		36	25.0	30.6	44.4	
6 (%)	154	32.5	28.6	39.0		185	36.8	35.1	28.1	
7 (%)	295	32.9	36.9	30.2		455	32.1	36.5	31.4	
8 (%)	225	34.2	24.9	40.9		387	32.8	36.7	30.5	
9 (%)	41	34.1	36.6	29.3		89	34.8	37.1	28.1	
>10 (%)	2	50.0	0.0	50.0		12	50.0	8.3	41.7	
**Ordering food frequency before lockdown**					0.279					0.402
Never (%)	226	28.8	27.9	43.4		416	30.5	36.3	33.2	
1–3 times a month (%)	415	34.5	32.0	33.5		643	33.7	35.8	30.5	
Once a week (%)	73	38.4	35.6	26.0		101	38.6	35.6	25.7	
Few times a week (%)	34	38.2	26.5	35.3		22	50.0	36.4	13.6	
Once a day (%)	1	0.0	100.0	0.0		2	100.0	0.0	0.0	
Several times a day (%)	2	50.0	50.0	0.0		4	25.0	50.0	25.0	
**Cooking habit before lockdown**					**0.007**					**<0.001**
I cook (%)	257	28.4	29.2	42.4		490	30.4	31.8	37.8	
Somebody cooks for me (%)	360	33.6	31.1	35.3		598	34.6	38.8	26.6	
Ordering or buying (%)	134	41.8	34.3	23.9		100	41.0	39.0	20.0	
**Consuming meals at regular times before lockdown**					0.252					0.341
Some of them (%)	389	31.6	33.7	34.7		638	33.1	36.7	30.3	
All of them (%)	83	27.7	31.3	41.0		167	28.1	35.9	35.9	
No (%)	279	37.3	27.2	35.5		383	36.3	34.7	29.0	
**Physical activity at work before lockdown**					**0.005**					**<0.001**
Low (%)	351	37.9	29.1	33.0		405	38.5	39.0	22.5	
Medium (%)	351	29.1	35.0	35.9		635	32.0	35.3	32.8	
High (%)	49	30.6	16.3	53.1		148	25.7	30.4	43.9	
**Physical activity during free time before lockdown**					**<0.001**					**<0.001**
Low (%)	221	42.1	29.9	28.1		320	40.0	37.8	22.2	
Medium (%)	379	31.9	35.1	33.0		660	34.1	35.0	30.9	
High (%)	151	23.8	22.5	53.6		208	21.2	36.1	42.8	

* *p* represents *p*-value between the groups evaluated by the χ^2^-test. *p*-values < 0.05 are highlighted in bold.

**Table 5:** Change of students’ lifestyle distributed according to levels of pHDI during the spring and winter lockdowns.

**Lifestyle Changes during the Lockdowns**	**pHDI**									
	**Spring Lockdown**					**Winter Lockdown**				
	All	Low	Medium	High	*p* *	All	Low	Medium	High	*p* *
	N	%	%	%		N	%	%	%	
	751	31.4	31.0	37.6		1188	39.9	32.7	27.4	
**Change of place of residence**					0.379					0.423
Yes (%)	408	30.9	29.4	39.7		261	36.8	35.6	27.6	
No (%)	343	32.1	32.9	35.0		927	40.8	31.8	27.4	
**Place of residence during lockdown**					0.957					0.095
Family home (%)	632	32.0	30.2	37.8		834	38.0	34.1	27.9	
Alone (%)	28	28.6	35.7	35.7		74	39.2	35.1	25.7	
Students’ residence (%)	34	26.5	38.2	35.3		119	39.5	34.5	26.1	
Shared flat (%)	57	29.8	33.3	36.8		161	50.3	23.0	26.7	
**Change of working status**					0.418					**0.002**
Don’t work, still employed (%)	49	34.7	30.6	34.7		20	55.0	25.0	20.0	
Lost my job (%)	89	32.6	23.6	43.8		93	31.2	23.7	45.2	
Working remotely (%)	44	36.4	38.6	25.0		43	39.5	23.3	37.2	
No change (%)	569	30.6	31.6	37.8		1032	40.4	34.0	25.6	
**Change of screen time**					0.711					**0.050**
Increased (%)	597	30.3	31.8	37.9		878	39.1	31.9	29.0	
Decreased (%)	25	40.0	28.0	32.0		23	21.7	39.1	39.1	
No change (%)	129	34.9	27.9	37.2		287	43.9	34.5	21.6	
**Change of sleeping time**					0.831					0.791
Increased (%)	429	30.5	32.6	36.8		449	40.1	31.0	29.0	
Decreased (%)	82	30.5	29.3	40.2		180	39.4	32.2	28.3	
No change (%)	240	33.3	28.8	37.9		559	39.9	34.2	25.9	
**Change of food ordering frequency**					**<0.001**					0.318
Increased (%)	52	59.6	21.2	19.2		203	44.8	30.0	25.1	
Decreased (%)	311	32.2	29.9	37.9		181	34.3	37.0	28.7	
No change (%)	388	27.1	33.2	39.7		804	39.9	32.3	27.7	
**Eating behavior**					0.707					0.259
Changed (%)	436	31.4	32.6	36.0		548	42.9	30.7	26.5	
Don’t know (%)	60	30.0	33.3	36.7		86	43.0	30.2	26.7	
No change (%)	255	31.8	27.8	40.4		554	36.5	35.0	28.5	
**Change of weight**					**0.009**					0.099
Increased (%)	235	38.7	31.1	30.2		329	43.8	28.6	27.7	
Decreased (%)	191	24.1	30.9	45.0		245	42.9	29.8	27.3	
No change (%)	325	30.5	31.1	38.5		614	36.6	36.0	27.4	
**Change of number of meals consumed**					0.064					**0.002**
Increased (%)	268	38.1	28.7	33.2		327	37.9	29.1	33.0	
Decreased (%)	102	26.5	31.4	42.2		188	50.5	29.3	20.2	
No change (%)	381	28.1	32.5	39.4		673	37.9	35.4	26.7	
**Change of cooking habits**					**0.001**					0.421
No change (%)	315	32.1	31.4	36.5		811	41.1	32.2	26.8	
Somebody started cooking for me (%)	209	34.9	31.6	33.5		118	34.7	38.1	27.1	
I started cooking (%)	217	24.0	31.3	44.7		200	36.0	34.0	30.0	
I started ordering food (%)	10	100.0	0.0	0.0		59	47.5	23.7	28.8	
**Consuming meals at regular times during lockdown**					**0.002**					**0.026**
Some of them (%)	386	28.8	34.5	36.8		553	37.1	35.4	27.5	
All of them (%)	149	24.2	30.2	45.6		163	34.4	32.5	33.1	
No (%)	216	41.2	25.5	33.3		472	45.1	29.4	25.4	
**Physical activity at work during lockdown**					**0.001**					**0.007**
Increased (%)	313	27.8	26.8	45.4		236	32.2	32.6	35.2	
Decreased (%)	270	31.9	37.4	30.7		623	43.8	31.9	24.2	
No change (%)	168	37.5	28.6	33.9		329	38.0	34.0	28.0	
**Physical activity during free time during lockdown**					**0.007**					**0.003**
Increased (%)	366	27.6	28.1	44.3		227	30.8	33.5	35.7	
Decreased (%)	235	36.2	33.2	30.6		612	44.3	31.5	24.2	
No change (%)	150	33.3	34.7	32.0		349	38.1	34.1	27.8	

* *p* represents *p*-value between the groups evaluated by the χ^2^-test. *p*-values < 0.05 are highlighted in bold.

## Data Availability

Data is contained within the article (or Supplementary Material).

## Supplementary Materials

The following supporting information can be downloaded at: https://www.mdpi.com/article/10.3390/foods12132532/s1, Table S1: Overview UNI-COVID questionnaire used to collect information on dietary and lifestyle behavior during two COVID-19 Lockdowns, Table S2: Students’ characteristics according to their fields of study.
