# Supplementary material for: Diet Quality and Its Association with Lifestyle and Dietary Behaviors among Croatian Students during Two COVID-19 Lockdowns
Source: Foods. 2023 Jun 29;12(13):2532. doi: 10.3390/foods12132532 (PMC10340265; doi:10.3390/foods12132532)
Supplement: Supplementary file 1 [file foods-12-02532-s001.zip › foods-2443866-supplementary.pdf]

foods

MDPI

Supplementary Material

The questionnaire in its entirety is available upon request at: Magdalena Czlapka-Matyasik, Poznan University of Life Sciences, Department of Human Nutrition and Dietetics, Poznan, Poland: magdalena.matyasik@up.poznan.pl

The basic sections of the questionnaire with related questions are listed in Table S1.

Table S1. Overview UNI-COVID questionnaire used to collect information on dietary and lifestyle behavior during two COVID-19 Lockdowns

**Section 1**
Agreed to participate in this research without any additional explanation, without any pressure, and consciously<br>Identified its field of study
**Section 2: Demographic**
Gender<br>Age<br>Weight & Height<br>Marital status<br>Place of residence (now & before social isolation period)<br>What is your graduated level of education<br>Working before social isolation period<br>Household’s overall situation<br>Hours/day spent in front of the screen (watching TV, using a computer, smart phone; before & during isolation)<br>During social isolation, have you changed: (i) your screen time; (ii) eating behavior; (iii) body weight & (iv) duration of sleeping
**Section 3: Eating habits**
Before and during isolation period: (i) Cooking; (ii) Frequency of ordering food; (iii) Number of consumed meals;<br>(iv) Consumption of listed food: Bread (any type); Rice, pasta, tortilla, cereals; Red meat (beef, pork); Processed meat (cured meat, smoked sausages, hot-dogs); White meat (turkey, chicken); Fish and seafood; Vegetables; Fruit; Pulses-based food (legume, chickpeas, beans); Dairy product (milk, yogurt, cheese); Egg; Sweet snacks; Savory snacks; Nuts, almonds, seeds; (v) consumption of drinks with sugar (added or sweetened); (vi) Consumption of listed drinks (Water; Tea; Coffee; Juice (fruit or vegetables); Sweetened carbonated beverage e.g., Cola, Pepsi; Unsweetened beverage; Energy drinks; Alcoholic beverage) and (vii) Physical activity
**Section 4: Psychological section**
Gathering a personal impression by checking the most appropriate statements to describe the participant

Table S2. Students' characteristics according to the fields of study.

Field of study		Age (mean ± SD)	BMI (mean ± SD)	Gender		
				Male (%)	Female (%)	Other (%)
Biotechnical	Spring	22.31 ± 2.59	22.06 ± 3.08	11.47	88.07	0.46
	Winter	21.10 ± 2.17	21.58 ± 2.70	9.19	88.65	2.16
Technical	Spring	22.43 ± 2.44	22.65 ± 3.15	27.91	72.09	0.00
	Winter	21.54 ± 2.13	22.93 ± 3.78	32.78	65.84	1.38
Social	Spring	22.52 ± 2.39	23.09 ± 4.01	6.48	93.52	0.00
	Winter	21.98 ± 2.46	21.77 ± 2.64	6.30	93.70	0.00
Biomedical	Spring	21.39 ± 2.14	22.17 ± 3.08	14.43	85.07	0.50
	Winter	21.23 ± 2.46	21.88 ± 3.88	11.35	88.30	0.35
Natural	Spring	21.51 ± 1.93	22.55 ± 3.98	15.07	82.19	2.74
	Winter	21.21 ± 2.17	22.50 ± 4.05	25.56	72.93	1.50
Humanistic	Spring	23.81 ± 3.32	22.68 ± 4.33	10.34	87.93	1.72
	Winter	21.91 ± 2.41	21.71 ± 2.89	7.69	90.77	1.54
Artistic	Spring	21.14 ± 2.12	24.59 ± 5.20	14.29	85.71	0.00
	Winter	20.61 ± 2.53	21.80 ± 2.86	3.57	92.86	3.57
